# Supplementary material for: The Assembly Process of Free-Living and Particle-Attached Bacterial Communities in Shrimp-Rearing Waters: The Overwhelming Influence of Nutrient Factors Relative to Microalgal Inoculation
Source: Animals (Basel). 2023 Nov 11;13(22):3484. doi: 10.3390/ani13223484 (PMC10668652; doi:10.3390/ani13223484)
Supplement: Supplementary file 1 [file animals-13-03484-s001.zip › animals-2646736-supplementary.pdf]

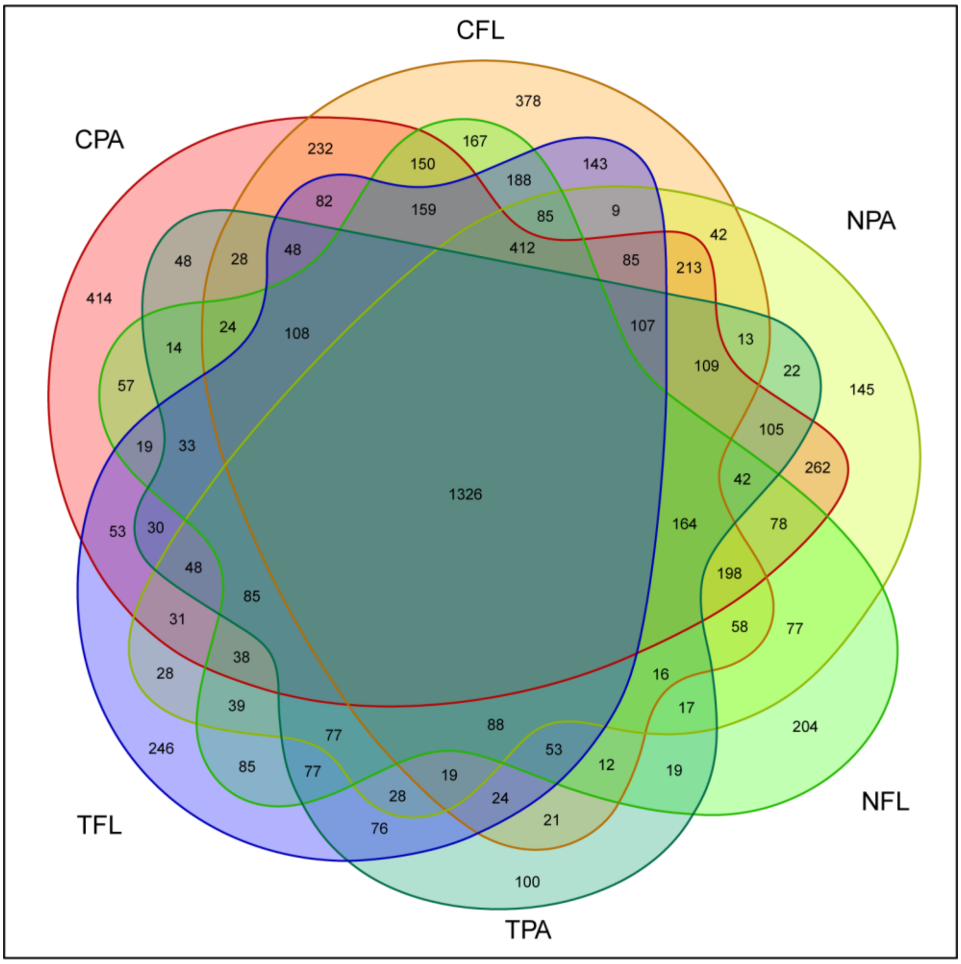

Figure S1. The number of ZOTUs in PA and FL bacterial communities among three groups.

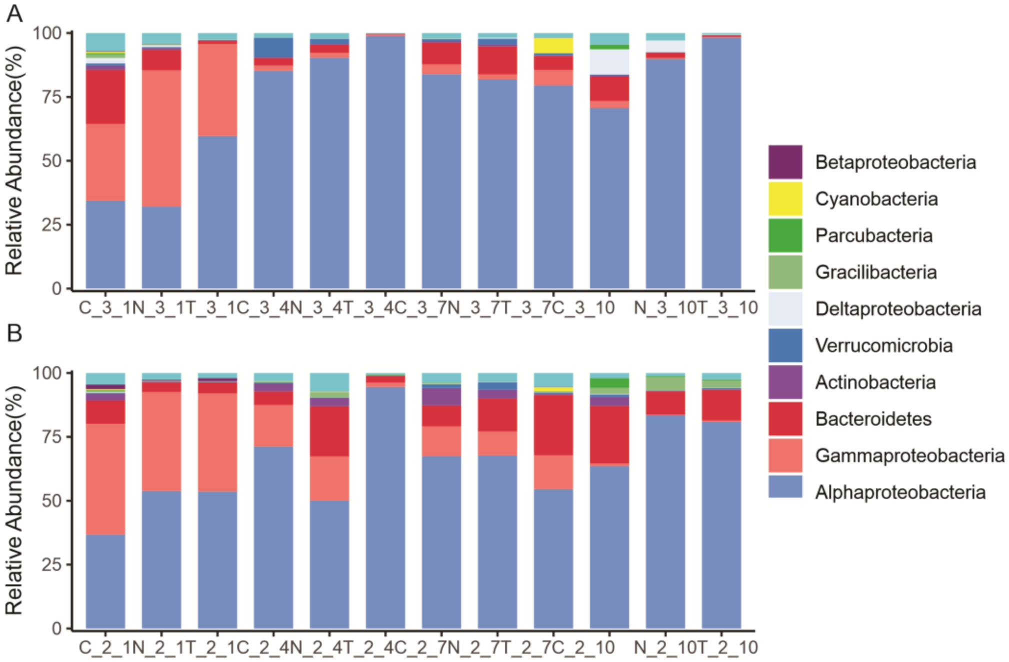

Figure S2. Taxonomic composition of particle-attached (**A**) and free-living (**B**) bacterial communities in shrimp-rearing water at phylum level (Proteobacteria phylum is grouped into the class level).

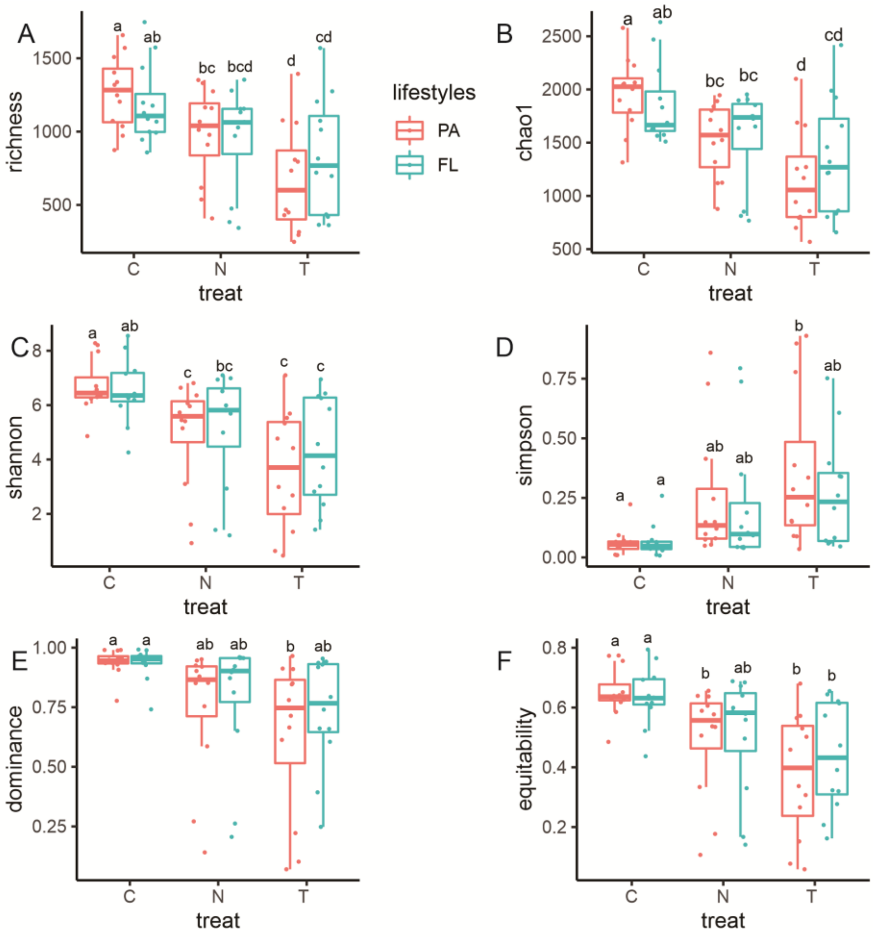

Figure S3. The α-diversity indices of particle-attached (PA) and free-living (FL) bacterial communities in three groups. (**A**) richness, (**B**) Chao1, (**C**) Shannon, (**D**) Simpson, (**E**) dominance, and (**F**) equitability. Different lowercase letters denote significant differences (p < 0.05) between rearing times based on one-way ANOVA.

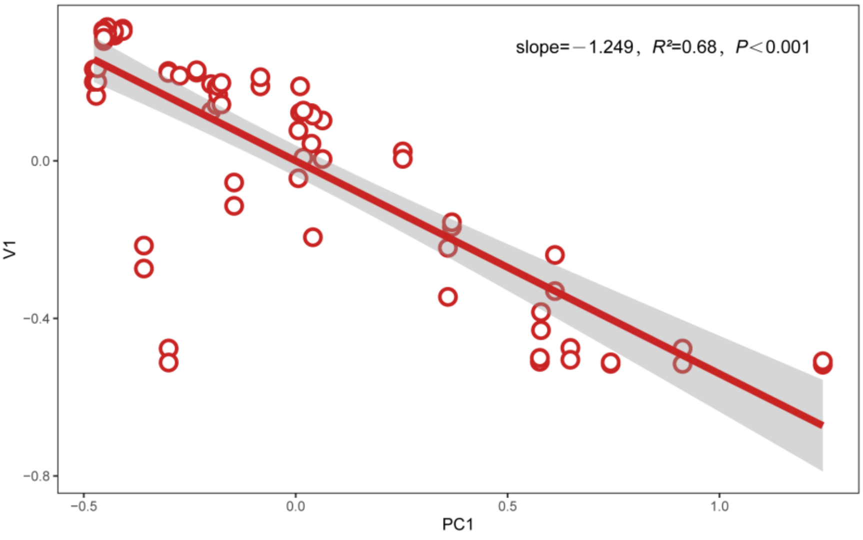

Figure S4. Regression analyses between the PCo 1 of PCoA and PC1 of PCA.

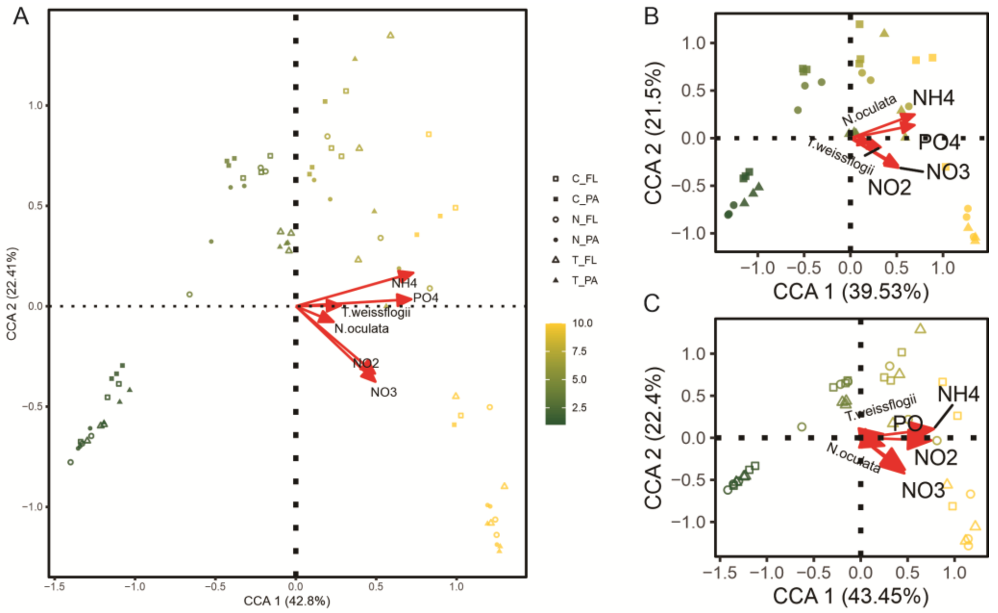

Figure S5. Driving effect of environment factors on whole (**A**), PA (**B**), and FL (**C**) bacterial communities visualized via canonical correspondence analysis (CCA).

Table S1. Spearman's correlation between the principal components (PC1 and PC2) of principal component analysis and environmental factors (nutrient and microalgal factors).

	PO_4	NH_4	NO_2	NO_3	*N. oculata*	*T. weissflogii*
PC1	0.974 ***	0.943 ***	0.654 ***	0.545 ***	0.358 **	−0.156
PC2	0.126	0.158	0.362 **	0.235 *	−0.806 ***	0.720 ***

* $p < 0.05$; ** $p < 0.01$; *** $p < 0.001$.

Table S2. Pairwise comparison of bacterial community of each group on the first day based on MRPP, ANOSIM, and Adonis.

	MRPP		ANOSIM		Adonis	
	δ	*p*	r	P	F. Model	*p*
C_PA/N_PA	0.51	0.1	1	0.1	20.25	0.1
C_PA/T_PA	0.61	0.1	1	0.1	37.19	0.1
N_PA/T_PA	0.46	0.1	1	0.1	14.93	0.1
C_FL/N_FL	0.35	0.1	1	0.1	7.96	0.1
C_FL/T_FL	0.39	0.1	1	0.1	10.04	0.1
N_FL/T_FL	0.13	0.1	0.44	0.1	2.75	0.1

Table S3. Spearman's correlation between the principal components (PC1 and PC2) of principal component analysis and α-diversity indices.

	Chao1	Dominance	Equitability	Richness	Simpson	Shannon
PC1	−0.241 *	−0.418 ***	−0.456 ***	−0.320 **	0.417 ***	−0.429 ***
PC2	−0.196	−0.245 *	−0.291 *	−0.246 *	0.243 *	−0.270 *

* $p < 0.05$; ** $p < 0.01$; *** $p < 0.001$.
